# Chest Wall Sarcoma: Outcome in 22 Patients After Resection Requiring Thoracic Cage Reconstruction

**DOI:** 10.1080/13577149877894

**Published:** 1998-12

**Authors:** Per Jönsson, Erik Gyllstedt, Göran Hambraeus, Ramon Lillogil, Anders Rydholm

**Affiliations:** 1 Department of Thoracic Surgery Lund University Hospital Lund S-22185 Sweden; 2 Department of Orthopaedics Lund University Hospital Lund S-22185 Sweden

## Abstract

*Purpose.* To evaluate the outcome after resection of malignant chest wall sarcoma, requiring reconstruction of the chest wall.

*Subjects.* Twenty-two patients, 15 with primary tumours, were operated on in our institution between 1983 and 1996. Four patients underwent surgery after a previous intralesional or marginal excision and three patients because of a local recurrence.

*Methods.* The tumour was resected ‘en bloc’, including skin, muscle and thoracic skeleton. When necessary, adjacent organs invaded by the tumour, such as lung, pericardium and diaphragm, were also removed to obtain a wide margin. Reconstruction of the chest wall was performed with Marlex mesh (n=9), methylmethacrylate cement (n=2) or a Marlex methylmethacrylate ‘sandwich’ (n=11).

*Results.* The median tumour size was 9.5 (2–20) cm. The most common type of tumour was chondrosarcoma (12 cases). No patient died in hospital. Five patients required reoperation because of complications, two patients because of loosening of the acrylate prosthesis, two because of necrosis of soft tissue coverage and one was reoperated because of bleeding. Four patients died of generalized tumour disease between 5 and 77 months after surgery and one patient died of a local recurrence 32 months after the primary operation. Seventeen patients are alive, with a median follow-up of 36 (4–162) months. Microscopic radicality (negative margin) was achieved in 17 patients but 5 of these had local recurrences. Two of five patients with positive margins had a local recurrence of the tumour. Of the seven patients with local recurrences, two also developed metastases.

*Discussion.* Large chest wall sarcomas can be successfully resected and the chest wall reconstructed with low morbidity and mortality.


*Sarcoma* (1998) **2**, 143–147

ORIGINAL ARTICLE

### Chest wall sarcoma: outcome in 22 patients after resection requiring thoracic cage reconstruction

PER JÖNSSON,[1] ERIK GYLLSTEDT,[1] GÖRAN HAMBRAEUS,[1] RAMON LILLOGIL[1] & ANDERS RYDHOLM[2]

[1]*Departments of Thoracic Surgery and* [2]*Orthopaedics, Lund University Hospital, S-22185 Lund, Sweden*

**Abstract**

*Purpose.* To evaluate the outcome after resection of malignant chest wall sarcoma, requiring reconstruction of the chest wall.

*Subjects.* Twenty-two patients, 15 with primary tumours, were operated on in our institution between 1983 and 1996. Four patients underwent surgery after a previous intralesional or marginal excision and three patients because of a local recurrence.

*Methods.* The tumour was resected 'en bloc', including skin, muscle and thoracic skeleton. When necessary, adjacent organs invaded by the tumour, such as lung, pericardium and diaphragm, were also removed to obtain a wide margin. Reconstruction of the chest wall was performed with Marlex mesh ($n = 9$), methylmethacrylate cement ($n = 2$) or a Marlex methylmethacrylate 'sandwich' ($n = 11$).

*Results.* The median tumour size was 9.5 (2–20) cm. The most common type of tumour was chondrosarcoma (12 cases). No patient died in hospital. Five patients required reoperation because of complications, two patients because of loosening of the acrylate prosthesis, two because of necrosis of soft tissue coverage and one was reoperated because of bleeding. Four patients died of generalized tumour disease between 5 and 77 months after surgery and one patient died of a local recurrence 32 months after the primary operation. Seventeen patients are alive, with a median follow-up of 36 (4–162) months. Microscopic radicality (negative margin) was achieved in 17 patients but 5 of these had local recurrences. Two of five patients with positive margins had a local recurrence of the tumour. Of the seven patients with local recurrences, two also developed metastases.

*Discussion.* Large chest wall sarcomas can be successfully resected and the chest wall reconstructed with low morbidity and mortality.

**Key words**: *chest wall, resection, reconstruction, sarcoma.*

### Introduction

In recent years, successful surgery has been reported even for advanced malignant chest wall tumours. Five-year survival rates between 20 and 90%[1–5] and local recurrence rates of 15–40%[3,6] have been achieved. The key to good long-term results seems to be extensive resection with a wide margin. Aggressive surgery is possible if chest wall repair affords enough stability and airtight closure. This may be obtained by reconstructing the thoracic cage with synthetic materials and appropriate soft tissue coverage, often with myo-cutaneous flaps. The present study reviews our experience with the surgical management of chest wall sarcoma requiring reconstruction of chest wall defects.

### Subjects and methods

Our University Hospital is a tertiary referral centre for patients with musculoskeletal tumours and serves about 1.5 million inhabitants. Between 1983 and 1996, we operated on 22 patients (13 men, 9 women) for primary or locally recurrent chest wall sarcoma requiring reconstruction of the chest wall. Patients with small sarcomas, where resection could be performed without chest wall repair, are excluded. The patients had a median age of 51 (16–82) years at the time of surgery. Diagnostic needle or incisional biopsy of the lesion was performed in all patients who had not previously undergone surgery, except patient no. 19. This patient had Mb Ollier and the diagnosis of secondary chondrosarcoma was obvious. Fourteen patients had a primary skeletal sarcoma and eight had a soft tissue sarcoma. The most common type of tumour was chondrosarcoma (12 cases). Two patients (nos. 5 and 21) developed a sarcoma at the site of previous tissue irradiation, having had a mastectomy 7 and

Correspondence to: P. Jönsson, Department of Thoracic Surgery, Lund University Hospital, S-22185 Lund, Sweden. Fax: 46-46-158 635.

1357-714X/98/030143–05 $9.00 © 1998 Carfax Publishing Ltd

14 years earlier. Seven patients had undergone limited intralesional or marginal local surgery in other institutions before referral. Three of them were referred for extended resection shortly after surgery, while the other four were referred after detection of a local recurrence. Three patients (with Ewing's sarcoma, osteosarcoma and malignant fibrous histiocytoma, respectively) received preoperative chemotherapy.

Preoperative evaluation of the tumour included chest roentgenography, computed tomography and, more recently, magnetic resonance imaging in four cases. Respiratory function was assessed with spirometry and, if needed, ventilation and perfusion scans and blood gases. Surgery was performed as a one-stage procedure. Profylactic antibiotic, cefuroxim, was administered prior to surgery. The tumour was resected '*en bloc*', including skin, muscle and thoracic skeleton, with an intended macroscopic tumour-free cuff. One normal rib above and one below the most cranial and caudal tumour extension were included in the specimen. In selected cases, resection included parts of the lung, the pericardium, the diaphragm and the abdominal wall. In two cases, the arm had to be sacrificed by a forequarter amputation. Depending on the location and size of the resected chest wall, reconstruction was performed with Marlex mesh or a Marlex methylmethacrylate 'sandwich'. Non-absorbable sutures of heavy calibre were used for fixation but for the sternum where stainless steel sutures were applied. If possible, soft tissue was closed *per primum*, otherwise myo-cutaneous flaps were used, in some cases with additional skin grafting. Two (nos. 4 and 14) of the five patients who did not obtain tumour-free margins were given postoperative radiotherapy.

All patients were followed regularly with a physical examination and chest radiographs. The median duration of follow-up for the surviving 17 patients is 36 (4–162) months.

### Results

The results are shown in Table 1. The median hospital stay was 9 (4–55) days. There was no perioperative mortality.

Five patients were reoperated because of complications. In two cases (patient nos. 5 and 6), the Marlex methylmethacrylate 'sandwich' loosened, due to ruptured sutures between the prosthesis and the ribs. The younger patient moved a great deal while awakening from the anaesthesia, which may have caused the loosening. She was reoperated on after 20 days and a new Marlex methylmethacrylate 'sandwich' was applied. The other patient also showed signs of early loosening of the prosthesis. Part of the circumference of this prosthesis was sutured to abdominal muscles. A conservative regime was chosen because of the patient's old age. However, skin and soft tissue necrosis developed and was followed by an infection. The prosthesis was removed and the infection healed. No reconstructive surgery has since been carried out and the patient now has an abdominal hernia, with mild discomfort.

Two patients developed soft tissue necrosis over the prosthesis after about two weeks. One patient (no. 21) required surgery in irradiated tissue (14 years after treatment for breast cancer) and was successfully reoperated with a myo-cutaneous rectus abdominis flap. The other patient (no. 18) with a generalized malignancy underwent a palliative primary resection due to a huge chest wall chondrosarcoma, causing severe symptoms and was reoperated with a new fascio-cutaneous flap, without further local complications.

Patient no. 17 (our first patient) was reoperated during the first postoperative night because of a haemorrhage. The source of bleeding was the left ventricle of the heart, where the acrylate prosthesis came in contact with the epicardium, causing an effect like sandpaper, since the pericardium had been removed *en bloc* with the tumour. The great omentum was interposed to remedy this.

Seven patients developed local recurrences, all confirmed by tissue samples, and 5 of these (nos. 8,12,19,21 and 22) were reoperated. Patient no. 8 is disease-free 9 months after the second operation and patient no. 12 after 78 months.

Five patients had metastases. Three of these had pulmonary metastases diagnosed already at the time of surgery. One patient (no. 5) is now being treated with chemotherapy but the other four have died.

Five patients (nos. 18–22) died of their malignancies between 5 and 77 months after the operation. Patient no. 21 died after a local recurrence and the other four because of metastatic disease. Another patient (no. 4) died in a car accident, with no detectable disease.

### Discussion

Sarcoma of the chest wall presents a therapeutic dilemma, if allowed to become large prior to surgery. A tumour-free margin for a chest wall sarcoma may require resection of several ribs and adjacent soft tissue and efforts must be made to restore chest wall stability and function. Reconstruction is essential for large anterior and lateral defects to prevent paradoxical respiration, but is generally not required for defects in the thoracic apex or sub-scapular region. A rigid chest wall prosthesis is important for preserving pulmonary function.[3,7] This makes early extubation possible and shortens hospitalization.[5,7] Myo-cutaneous flaps can be used without rigid support, but the risk of prolonging postoperative ventilatory support increases.[7] These flaps may also become more flaccid with time.

Many synthetic and biological materials have been used over the years, including Marlex mesh,

**Table 1**. *Patient data*

| Pat. | Age (years) | Sex | Operation | Resected tissue |
|---|---|---|---|---|
| 1 | 18 | m | primary | costae VII–IX, pulmonary wedge |
| 2 | 51 | f | primary | costae V–VIII, part of sternum |
| 3 | 45 | f | recurrent | clavicle, costa I, part of sternum |
| 4 | 24 | m | primary | costae I–II |
| 5 | 61 | f | primary | costae III–VI |
| 6 | 82 | m | primary | costae VII–IX, part of diaphragm |
| 7 | 47 | m | primary | costae IV–VI, part of sternum |
| 8 | 40 | f | extended | costae Vl–VIII |
| 9 | 34 | m | extended | costae II–IV |
| 10 | 65 | m | primary | costa IV |
| 11 | 38 | f | primary | costae II–IV, part of sternum |
| 12 | 38 | m | primary | costae III–V |
| 13 | 54 | f | recurrent | sternum |
| 14 | 16 | m | primary | costa IV |
| 15 | 74 | f | primary | costae IV–VI |
| 16 | 77 | f | extended | costae VII–VIII |
| 17 | 56 | m | primary | part of sternum |
| 18 | 67 | m | recurrent | sternum, costal arci |
| 19 | 51 | m | primary | c. IV–IX, peric., diaphr., abd. wall, pulm. wedge |
| 20 | 39 | m | recurrent | fqa, muscle overpreviously resected ribs |
| 21 | 66 | f | primary | fqa, c. II–IV, part of sternum |
| 22 | 51 | m | primary | costal X–XI, part of diaphragm |

| Pat. | Reconstruction | Soft tissue coverage | Type of sarcoma—grade | Tumour size (cm) |
|---|---|---|---|---|
| 1 | acrylate/marlex | direct closure | osteosarcoma—H | 10 |
| 2 | acrylate | direct closure | malignant schwannoma—H | 4 |
| 3 | marlex | direct closure | chondrosarcoma—L | 4 |
| 4 | marlex | direct closure | spindle cell sarcoma—H | 10 |
| 5 | acrylate/marlex | m. latissimus flap + skin graft | spindle cell sarcoma—H | 8 |
| 6 | acrylate | direct closure | chondrosarcoma—H | 9 |
| 7 | acrylate/marlex | direct closure | chondrosarcoma—H | 6 |
| 8 | acrylate/marlex | direct closure | synovial sarcoma—H | 10 |
| 9 | acrylate/marlex | direct closure | malignant schwannoma—L | 20 |
| 10 | marlex | direct closure | chondrosarcoma—L | 3 |
| 11 | acrylate/marlex | direct closure | chondrosarcoma—H | 9 |
| 12 | marlex | direct closure | chondrosarcoma—H | 12 |
| 13 | acylate/marlex | direct closure | chondrosarcoma—L | 2 |
| 14 | marlex | direct closure | Ewing's sarcoma—H | 9 |
| 15 | marlex | direct closure | chondrosarcoma—L | 7 |
| 16 | marlex | direct closure | liposarcoma—H | 5 |
| 17 | acrylate/marlex | myo-cutaneous flap | chondrosarcoma—H | 13 |
| 18 | marlex | myo-cutaneous flap + skin graft | chondrosarcoma—H | 10 |
| 19 | acrylate/marlex/Goretex | m. latissimus flap + skin graft | chondrosarcoma—H | 20 |
| 20 | acrylate/marlex | cutaneous flap | fibrosarcoma—H | 12 |
| 21 | acrylate/marlex | cutaneous flap | malign. fibrous histiocytoma—H | 12 |
| 22 | marlex | direct closure | chondrosarcoma—H | 13 |

methylmethacrylate, Goretex, Vicryl, bone, metal, fascia and dehydrated human dura.[3,9–13] Some have been abandoned—for example, fascia lata, due to a tendency to become flaccid with time.[11] We used Marlex mesh or a stiff prosthesis made of methylmethacrylate (bone cement). Methylmethacrylate sandwiched between two layers of Marlex mesh weighs little and is versatile, bonds firmly to bone, allows tissue in-growth, and is radiolucent for subsequent lung-field evaluation.[10] In our hands, bone cement was of particular value when an arched prosthesis for the lateral thoracic wall was required. We have had two cases of loosening of the prosthesis. Stable anchorage to the ribs or sternum is obviously important. We have preferred to limit the size of the prosthesis to prevent the edge of the

**Table 1.** *Patient data—continued*

| Pat. | Tumour-free margin | Hospital stay (days) | Follow-up (months) | Local recurrence (months) | Metastasis (months) |
|---|---|---|---|---|---|
| 1 | yes | 5 | 4 | | |
| 2 | yes | 7 | 7 | | |
| 3 | yes | 7 | 11 | | |
| 4 | no | 6 | 12 | | |
| 5 | yes | 55 | 17 | 12 | 12 |
| 6 | yes | 12 | 19 | | |
| 7 | yes | 6 | 24 | | |
| 8 | yes | 4 | 32 | 23 | |
| 9 | yes | 9 | 36 | | |
| 10 | yes | 6 | 70 | | |
| 11 | yes | 10 | 111 | | |
| 12 | yes | 11 | 120 | 43 | |
| 13 | yes | 28 | 121 | | |
| 14 | no | 9 | 126 | | |
| 15 | yes | 7 | 127 | | |
| 16 | yes | 7 | 127 | | |
| 17 | yes | 25 | 162 | | |
| 18 | no | 21 | 5* | | 0 |
| 19 | no | 20 | 13* | 6 | 0 |
| 20 | yes | 27 | 25* | 12 | 12 |
| 21 | yes | 38 | 32* | 5 | |
| 22 | no | 7 | 77* | 31 | 0 |

Pat. = patient number; age = age at the time of surgery; f = female; m = male; primary = previously not operated; extended = reoperation of primary tumour; previous operation considered not radical; recurrent = local recurrent sarcoma; reconstruction = type of prosthesis used; fqa = fore-quarter amputation; Goretex used to replace resected diaphragm; H = high-grade sarcoma; L = low-grade sarcoma; tumour size: largest diameter; follow-up: last five patients deceased*; metastasis 0 = metastasis present at time of surgery.

prosthesis from riding on an edge of the rib or sternum.

Infection of the prosthesis might be a problem.[6] An overall wound infection rate of about 5% has been reported by several authors. If the soft tissue, overlying the prosthesis, is inadequately vascularized, the infection rates increases.[5,10,14] We experienced only one patient with infection and this occurred after soft tissue necrosis secondary to the pressure of a loose prosthesis. We have used gentamicin in the cement which may have had a positive effect.

It is often possible to create soft tissue coverage, by direct closure or by using local myo-cutaneous flaps. Primary closure was done in 16 of our patients having deep-seated tumours with no involvement of the skin. However, in some cases, with large defects or damaged tissue due to prior radiation therapy, more distant muscle must be transposed.[15] We had one case with necrosis of the myo-cutaneous flap in tissue previously radiated because of breast cancer. This patient healed after a reoperation with transposition of the rectus abdominis muscle.

Five patients had positive microscopic margins and two of them developed local recurrences. Three of the five patients died during follow-up because of their malignancies and another patient was killed in a car accident one year after the operation, without known recurrent disease. The fifth patient, with Ewing's sarcoma, received adjuvant chemotherapy and is free from malignant disease, more than 10 years after surgery. Different combinations of surgery, chemotherapy and radiotherapy are employed for musculoskeletal sarcomas. Osteosarcoma (including malignant fibrous histiocytoma of bone) and Ewing's sarcoma are commonly treated by preoperative and postoperative chemotherapy whereas chemotherapy for soft tissue sarcomas is controversial.[16] Local radiotherapy, pre- or postoperatively, reduces the local recurrence risk especially when there is tumour in the resection border. The only independent poor-risk factor known to cause a local recurrence is incomplete tumour resection.[4,17] Distant metastases of chondrosarcoma occur in one third of patients who develop local recurrences, but in only 4% of those without local recurrences.[18]

Our results provide evidence that a large malignant tumour of the chest wall can be successfully managed with low morbidity and mortality by wide excision and reconstruction of the chest wall.

### References

1 Gordon MS, Hajdu SI, Bains MS, Burt ME. Soft tissue sarcomas of the chest wall. Results of surgical resection. *J Thorac Cardiovasc Surg* 1991; 101:843–54.

2 Burt M, Fulton M, Wessner-Dunlap S, Karpeh M, Huvos AG, Bains MS, Martini N, McCormack PM, Rusch VW, Ginsberg RJ. Primary bony and cartilaginous sarcomas of chest wall: results of therapy. *Ann Thorac Surg* 1992; 54:226–32.

3 Andersson BO, Burt ME. Chest wall neoplasms and their management. *Ann Thorac Surg* 1994; 58:1774–81.
4 Cakir S, Dincbas FO, Uzel O, Koca SS, Okkan S. Multivariate analysis of prognostic factors in 75 patients with soft tissue sarcoma. *Radiother Oncol* 1995; 37:10–16.
5 Martini N, Huvos AG, Burt ME, Heelan RT, Bains MS, McCormack PM, Rusch VW, Weber M, Downey RJ, Ginsberg RJ. Predictors of survival in malignant tumours of the sternum. *J Thorac Cardiovasc Surg* 1996; 111:96–106.
6 Chapelier A, Macchiarini P, Rietjens M, Lenot B, Margulis A, Petit JY, Dartevelle P. Chest wall reconstruction following resection of large primary malignant tumours. *Eur J Cardio-thorac Surg* 1994; 8:351–7.
7 Nash AG, Tuson JRD, Andrews SM, Stacey-Clear A. Chest wall reconstruction after resection of recurrent breast tumours. *Ann Royal Col Surg Engl* 1991; 73:105–9.
8 Kroll SS, Walsh G, Ryan B, King RC. Risks and benefits of using Marlex mesh in chest wall reconstruction. *Ann Plast Surg* 1993; 31:303–6.
9 Graham J, Usher FC, Perry JL, Barkley HT. Marlex mesh as a prosthesis in the repair of thoracic wall defects. *Ann Surg* 1960; 151:469–78.
10 McCormack P, Bains M, Beattie EJ, Martini N. New trends in skeletal reconstruction after resection. *Ann Thorac Surg* 1981; 31:45–52.
11 McCormack PM, Bains MS, Martini N, Burt ME, Kaiser LR. Methods of skeletal reconstruction following resection of lung carcinoma invading the chest wall. *Surg Clin N Am* 1987; 67:979–86.
12 Lampl LH, Loeprecht H. Chest wall resection–alloplastic replacement. *Thorac Cardiovasc Surgeon* 1988; 36:157–8.
13 Walton JM, Bass J, Sambey E, Rubin SZ. Use of human dura in pediatric chest wall reconstruction after tumour resection. *J Pediatr Surg* 1994; 29:1189–91.
14 Eng J, Sabanathan S, Pradhan GN, Mearns AJ. Primary bony chest wall tumours. *J R Coll Edinb* 1990; 35:44–7.
15 Pairolero PC, Arnold PG. Thoracic wall defects: surgical management of 205 consecutive patients. *Mayo Clin Proc* 1986; 61:557–63.
16 Thierney JT. Adjuvant chemotherapy for localised resectable soft tissue sarcoma of adults: a meta-analysis of individual patient data. *The Lancet* 1997; 350:1647–54.
17 McAfee MK, Pairolero PC, Bergstralh EJ, Piehler JM, Unni KK, McLeod RA, Bernatz PE, WS Payne. Chondrosarcoma of the chest wall: factors affecting survival. *Ann Thorac Surg* 1985; 40:535–41.
18 Burt M. Primary malignant tumours of the chest wall: the Memorial Sloan–Kettering Cancer Center experience. *Chest Surg Clin N Am* 1994; 4:137–54.
